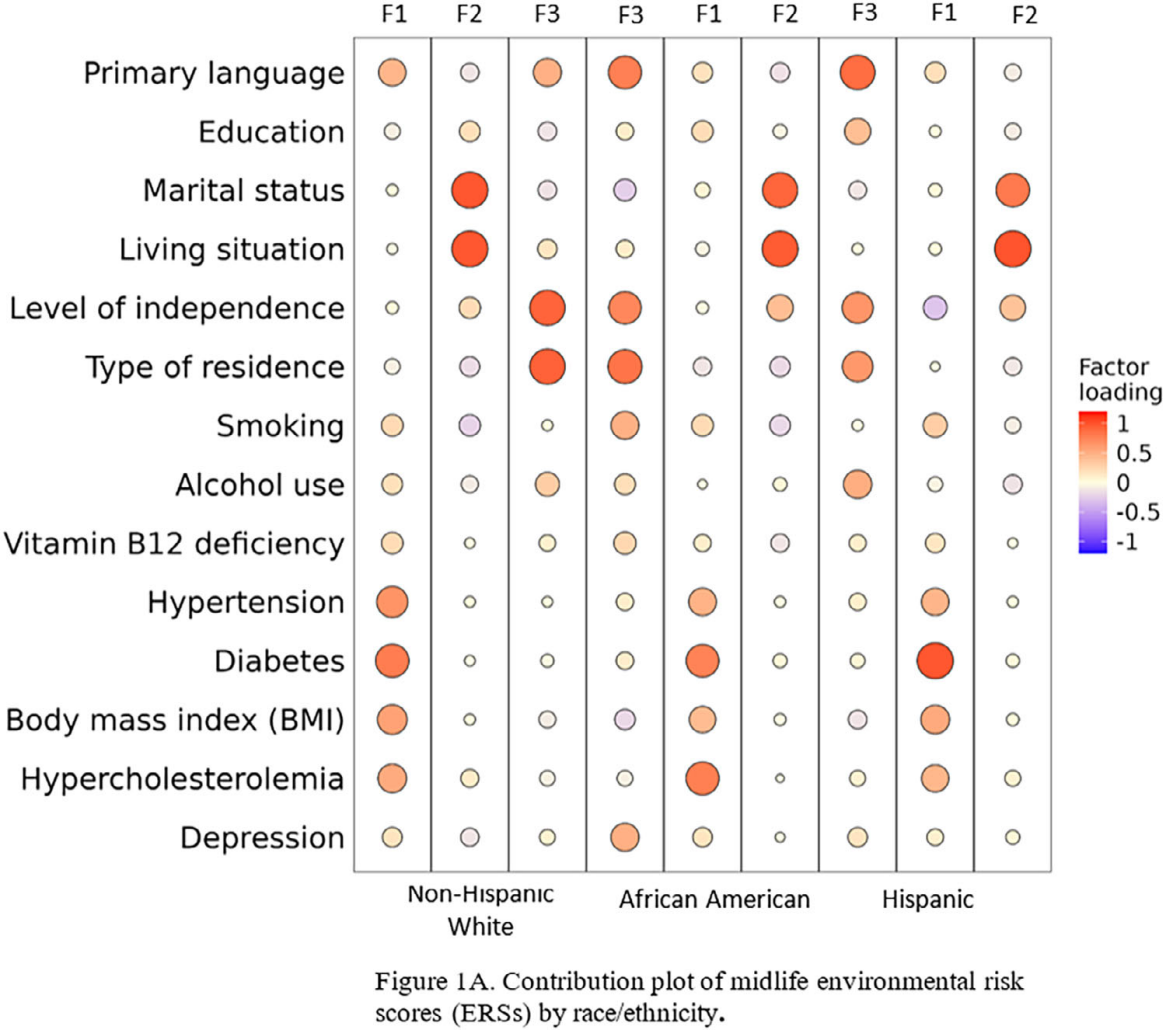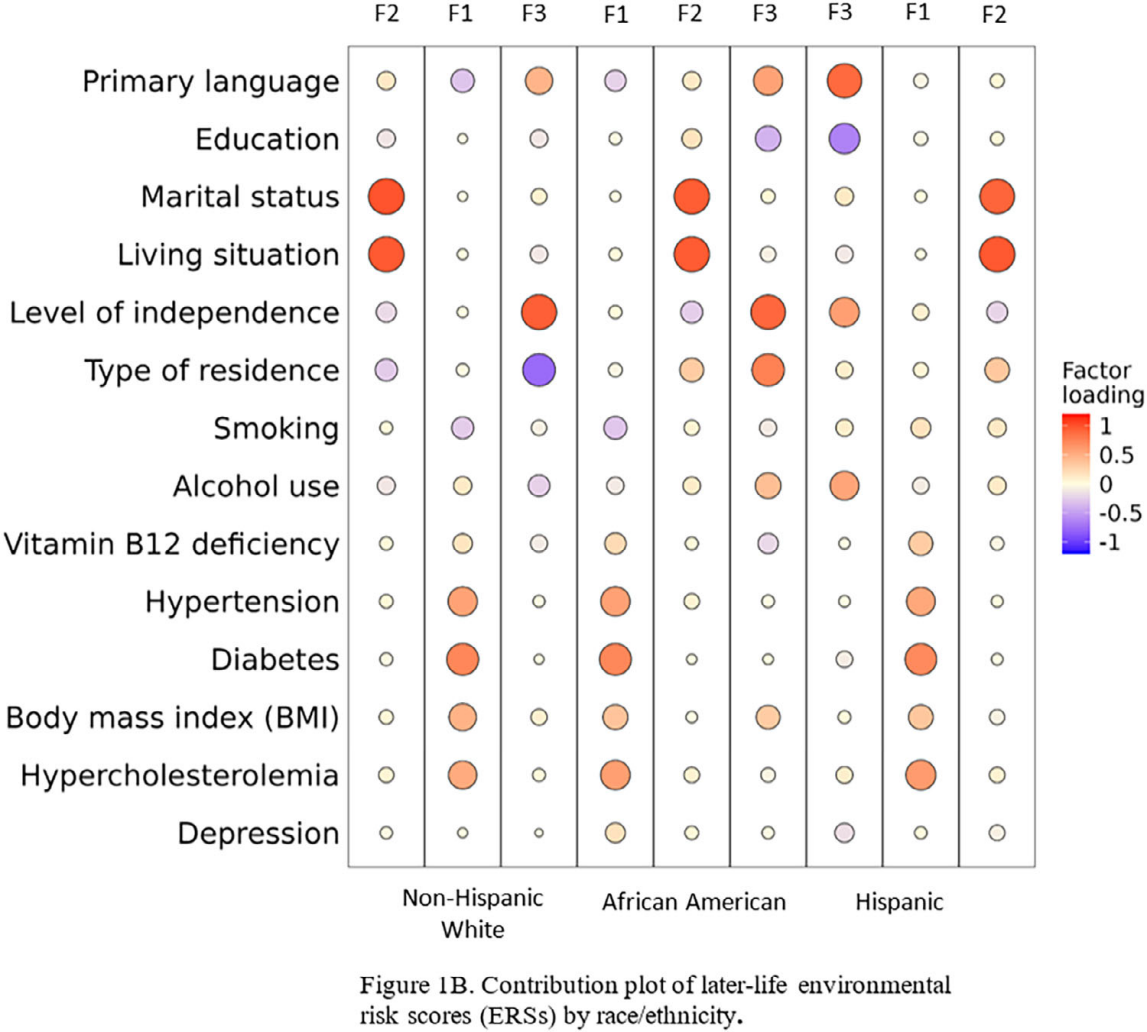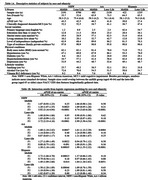# Midlife and Later‐Life Environmental Risk Scores (ERSs) Modify the Associations of Apolipoprotein E (*APOE*) ε4 with Dementia

**DOI:** 10.1002/alz70861_108987

**Published:** 2025-12-23

**Authors:** Xian Wu, Inori Tsuchiya, Khine Zin Aung, Qi Qiao, Xiaotong Ning, David W. Fardo, Erin L. Abner, Peter T. Nelson, Bryan D James, Yuriko Katsumata

**Affiliations:** ^1^ Department of Biostatistics, College of Public Health, University of Kentucky, Lexington, KY USA; ^2^ Sanders‐Brown Center on Aging, University of Kentucky, Lexington, KY USA; ^3^ College of Public Health, University of Kentucky, Lexington, KY USA; ^4^ Department of Epidemiology and Environmental Health, University of Kentucky, Lexington, KY USA; ^5^ Department of Pathology, University of Kentucky, Lexington, KY USA; ^6^ Department of Internal Medicine Rush University Medical Center, Chicago, IL USA; ^7^ Rush Alzheimer's Disease Center, Chicago, IL USA

## Abstract

**Background:**

Genetic factors contribute to the development of dementia, while genetic risk is modified by environmental influences. This study aimed to apply item response theory (IRT) to estimate environmental risk scores (ERSs) and investigate their interactions with *APOE* ε4 carrier status in dementia risk across the lifespan and diverse populations.

**Method:**

Data were drawn from the National Alzheimer’s Coordinating Center (NACC) Uniform Data Set (UDS) June 2024 data freeze. Using social determinants, physical conditions, and lifestyle variables, we applied an IRT‐based multidimensional generalized partial credit model (GPCM) to generate midlife (aged 40‐64) and later‐life (aged ≥ 65) ERSs for three ethnoracial groups: Non‐Hispanic White (NHW), African American (AA), and Hispanic. “Present” was assigned to physical conditions if subjects’ records indicate their present at least once during the visits, while other measures available at the most recent or initial visit were selected. Higher ERSs indicated greater environmental risk. Clinically diagnosed dementia or mild cognitive impairment (MCI) were the phenotype. Within each group, logistic regression was employed to examine interactions, adjusting for age and sex.

**Result:**

Sample sizes ranged from 422 (midlife Hispanic) to 8,706 (later‐life NHW). *APOE* ε4 carriers ranged from 27.4% to 46.5%, while subjects with dementia or MCI were 34.7% ∼ 52.5%. Comparative fit index (CFI > 0.90) and root mean square error of approximation (RMSEA < 0.04) indicated a three‐factor GPCM model was adequate and can be nominally described as one physical factor (F1) and two social factors focusing on marital status and level of independence (F2 and F3, respectively). F3 score interplayed with *APOE* ε4 across lifespan and ethnoracial groups. Midlife F1 score was associated with increased dementia or MCI risk in NHW *APOE* ε4 no‐carriers. Later‐life F2 score in the AA *APOE* ε4 no‐carriers group and midlife F2 scores in both Hispanic *APOE* ε4 carrier and non‐carrier groups were associated with elevated risk of cognitive impairment.

**Conclusion:**

Associations of *APOE* were modified by midlife and later‐life ERSs with dementia or MCI across diverse populations. Future research should include additional social and lifestyle measures, more genetic factors, consider the timing of the exposure measurements, and other phenotypes.